# p53 induces miR199a-3p to suppress SOCS7 for STAT3 activation and renal fibrosis in UUO

**DOI:** 10.1038/srep43409

**Published:** 2017-02-27

**Authors:** Ruhao Yang, Xuan Xu, Huiling Li, Jinwen Chen, Xudong Xiang, Zheng Dong, Dongshan Zhang

**Affiliations:** 1 Department of Emergency Medicine, Second Xiangya Hospital, Central South University, Changsha, Hunan 410011, China.; 2 Department of Emergency Medicine, Renmin Hospital of Wuhan University, Wuhan, Hubei, P.R. China; 3 Department of Ophthalmology, Second Xiangya Hospital, Central South University, Changsha, Hunan, People’s Republic of China; 4 Department of Nephrology, Second Xiangya Hospital, Central South University, Changsha, Hunan 410011, China.; 5 Department of Cellular Biology and Anatomy, Medical College of Georgia at Augusta University and Charlie Norwood VA Medical Center, Augusta, GA, USA

## Abstract

The role of p53 in renal fibrosis has recently been suggested, however, its function remains controversial and the underlying mechanism is unclear. Here, we show that pharmacological and genetic blockade of p53 attenuated renal interstitial fibrosis, apoptosis, and inflammation in mice with unilateral urethral obstruction (UUO). Interestingly, p53 blockade was associated with the suppression of miR-215-5p, miR-199a-5p&3p, and STAT3. In cultured human kidney tubular epithelial cells (HK-2), TGF-β1 treatment induced fibrotic changes, including collagen I and vimentin expression, being associated with p53 accumulation, p53 Ser15 phosphorylation, and miR-199a-3p expression. Inhibition of p53 by pifithrin-α blocked STAT3 activation and the expression of miR-199a-3p, collagen I, and vimentin during TGF-β1 treatment. Over-expression of miR-199a-3p increased TGFβ1-induced collagen I and vimentin expression and restored SOCS7 expression. Furthermore, SOCS7 was identified as a target gene of miR-199a-3p, and silencing of SOCS7 promoted STAT3 activation. ChIp analyses indicated the binding of p53 to the promoter region of miR-199a-3p. Consistently, kidney biopsies from patients with IgA nephropathy and diabetic nephropathy exhibited substantial activation of p53 and STAT3, decreased expression of SOCS7, and increase in profibrotic proteins and miR-199a-3p. Together, these results demonstrate the novel p53/miR-199a-3p/SOCS7/STAT3 pathway in renal interstitial fibrosis.

Renal interstitial fibrosis, characterized by excessive accumulation of extracellular matrix (ECM), leads to progressive decline in renal function and is a common pathological process of chronic kidney disease (CKD)^1^,^2^. The pathogenesis of renal interstitial fibrosis includes inflammatory response, proliferation of myofibroblasts, tubular epithelial cell injury, loss of capillary integrity, and related molecular factors^3^,^4^. p53 has been suggested to play a role in the renal fibrosis^5^,^6^,^7^,^8^,^9^,^10^,^11^,^12^. In 2010, Yang *et al*. reported that p53 activation in response to acute and chronic renal injury resulted in the progression of renal fibrotic diseases^5^. Pifithrin-α, a pharmacological p53 inhibitor, has been suggested to ameliorate profibrotic genes by inhibiting connective tissue growth factor (CTGF) and plasminogen activator inhibitor-1 (PAI-1) in rat renal fibroblast cells and human renal tubular epithelial cells (HK-2)^7^,^8^,^9^. Furthermore, Ying *et al*. reported that the deletion of p53 in proximal tubule cells prevented interstitial fibrogenesis after acute kidney injury (AKI) in mice^10^. However, Dagher *et al*.^11^ reported that pifithrin-α promoted renal fibrosis after initial injury in rat AKI. Fukuda *et al*.^12^ also demonstrated that renal fibrosis was enhanced in both global and podocyte-specific p53 knockout mice with Alport syndrome (AS). These results indicate that p53 has distinct or opposite roles in the pathogenesis of renal fibrosis in different types of cells, tissues or disease models, simultaneously, the role and molecular mechanism of p53 in renal fibrosis remains unclear.

Our current study was designed to investigate the role of p53 in UUO-associated renal fibrosis by using pharmacological and genetic inhibitory approaches. Moreover, we investigated the mechanism whereby p53 contributed to the renal fibrosis. We demonstrated that blockade of p53 led to the attenuation of renal fibrosis in mice with UUO, supporting a fibrogenic role of p53. We further showed that p53 may induce miR199a-3p to suppress SOCS7 for STAT3 activation, subsequently resulting in renal fibrosis.

## Results

### p53 was induced in UUO mice

**Figure 1:**
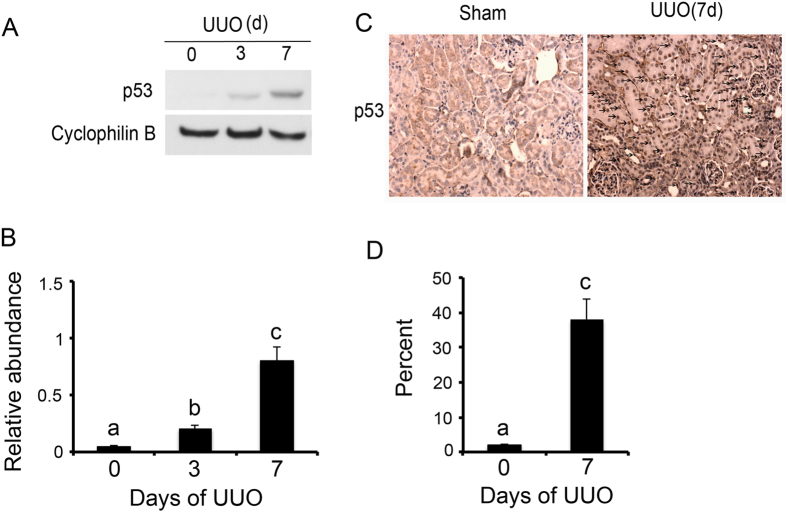
p53 was induced in UUO mice. The left ureter of male C57 mice was ligated for 0–7 days. (**A**) Immunoblot analysis of p53 and cyclophilin B (loading control) from mice kidneys of UUO groups at indicated time points. (**B**) Densitometric analyses were performed from each group (n = 8). (**C**) Immunohistochemical staining of p53 in kidney cortical tissues of sham and UUO mice at 7 days. (**D**) Representative quantification of p53 staining. Data were expressed as means ± sd (n = 8). Black arrows indicated the positive immunohistochemical staining of p53; ^#^*p* < *0.05*: day 3 or day 7 vs day 0; *p < *0.05*: day 7 vs day 3. Original magnification, x200.

Firstly, we analyzed p53 expression in kidney tissues with UUO at different time points. The p53 expression was very low in sham control (day 0) but upregulated significantly after UUO at day 3 & 7 (Fig. 1A). The immunohistochemical results further indicated that p53 was mainly expressed in the renal cortex from UUO mice at day 7. These data, confirming previous studies^7^, indicated the induction of p53 in UUO mouse model.

### Inhibition of p53 activity attenuated renal fibrosis and apoptosis by pifithrin-α in UUO mice

**Figure 2:**
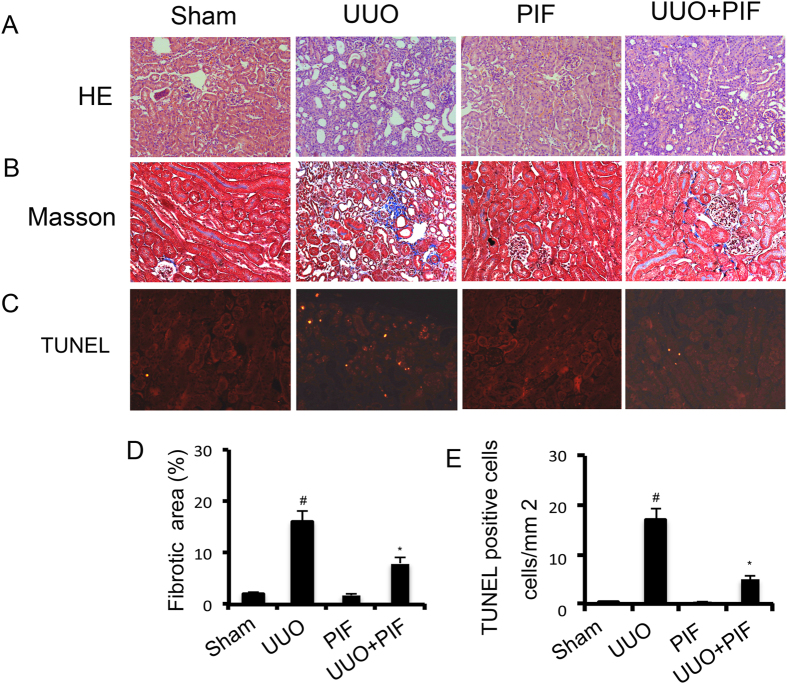
Pifithrin-α attenuated renal fibrosis and apoptosis in UUO mice. Male C57 mice after UUO were injected with 3 mg/kg pifithrin-α for 7 days. As controls, mice of the sham group were injected with saline. Renal cortical tissues were collected for hematoxylin and eosin staining to examine histology (**A**), Masson staining for fibrosis analysis (**B**), and for TUNEL assay of apoptosis (**C**). Tubulointerstitial fibrosis (**D**) and apoptosis (**E**) in the kidney cortex were quantified. Data were expressed as means ± sd (n = 8); ^#^*p* < *0.05*: the UUO group vs the sham group; *p < *0.05*: the PIF group vs the UUO group. Original magnification, x200 or 400.

Recent researches suggested that p53 may have distinct roles in the regulation of fibrosis in different animal models^5^,^6^,^7^,^8^,^9^,^10^,^11^,^12^. In the UUO kidneys, we observed significant tubular dilation and atrophy on day 7; however, it was notably reduced following pifithrin-α treatment (Fig. 2A). To assess the role of p53 in the pathogenesis of renal fibrosis, we determined the effect of pharmacologic inhibition of p53 in UUO mice. The expression of interstitial collagen fibrils was examined by the Masson trichrome staining in kidneys of the male C57 mice after UUO injury. As shown in Fig. 2B, kidneys with UUO for 7 days showed interstitial expansion of collagen accumulation and deposition, demonstrated by an increase in the Masson trichromepositive areas within the tubulointerstitium. However, it was markedly attenuated in UUO mice with pifithrin-α treatment (Fig. 2B). A quantitative analysis of the Masson trichrome-positive areas also demostrated similar results (Fig. 2D). Previous results reported that p53 inhibition would reduce apoptosis of tubular cells in AKI^13^,^14^. In the present study, we tried to determine whether p53 inhibition also suppresses apoptosis of tubular cells in UUO mice. Moreover, apoptotic cells, revealed by the TUNEL assay, were rarely seen in the sham and pifithrin-α control kidney tissues. After UUO injury, the number of apoptotic cells increased to 17/mm^2^ in the cortical tissue of UUO mice, but only to 4/mm^2^ in the cortical tissue in the pifithrin-α treatment group (Fig. 2C,E).

### Inhibition of p53 suppressed ECM accumulation by blocking STAT3 activation in UUO mice

**Figure 3:**
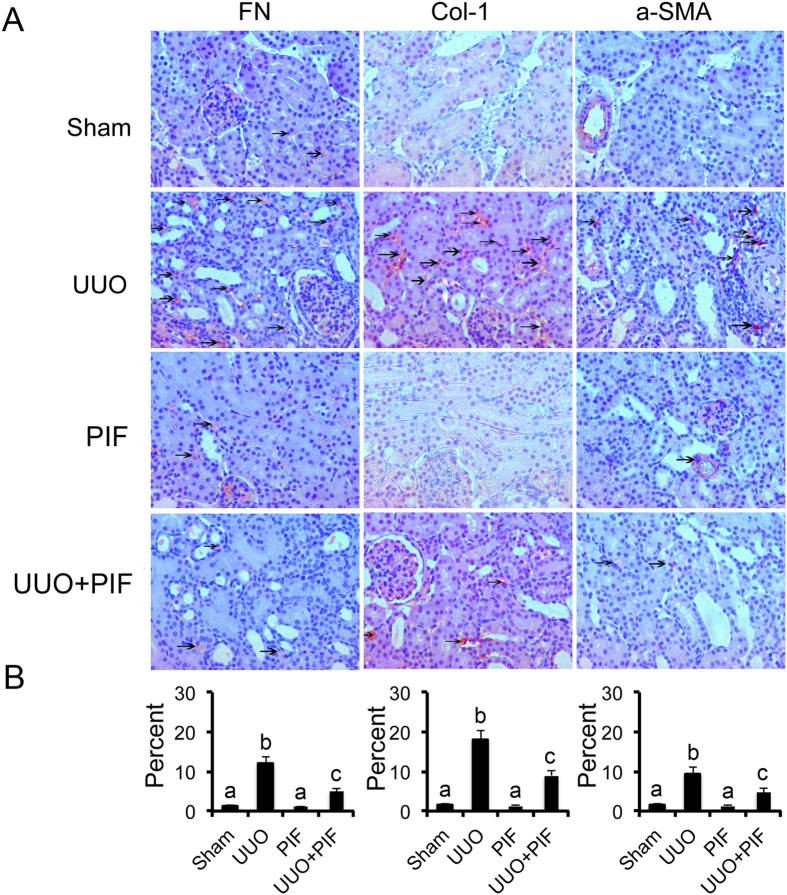
Pifithrin-α inhibited the expression of fibronectin, collagen I, and α-SMA by blocking STAT3 signaling in UUO mice. Male C57 mice after UUO were injected with 3 mg/kg pifithrin-a for 7 days. As controls, mice of the sham group were injected with saline. Renal cortical tissues were collected for immunohistochemistry staining to examine the expression of fibronectin, collagen I and α-SMA (**A**) and quantify immunohistochemistry staining (**B**). Data were expressed as means ± sd (n = 8); The black arrows indicated the positive immunohistochemical staining of fibronectin, collagen I, and α-SMA. ^#^*p* < *0.05*: the UUO group vs the sham group; *p < *0.05*: the PIF group vs the UUO group. Original magnification, x200.

Previous results demonstrated that STAT3 mediated renal fibrosis^15^,^16^,^17^. Recent studies reported that p53 inhibition blocked transient DOX-induced STAT3 activation in MHC-CB7 mice^18^. We hypothesized that inhibition of p53 suppressed renal interstitial fibroblasts by inhibiting STAT3 activation. As such, we examined the effect of pifithrin-α on the activation of p53 and STAT3 as well as the expression of fibronectin, α-SMA, and collagen I in UUO model. By immunohistochemistry, we found that the expression of fibronectin, collagen I, and α-SMA were markedly increased in UUO mice on day 7. In contrast, in the Sham group, they were significantly reduced following pifithrin-α treatment (Fig. 3A,B). Importantly, p53 accumulation and phosphorylation at serine-15 induced by UUO injury was suppressed by pifithrin-α treatment (Supplementary Figure 1). Interestingly, STAT3 tyrosine phosphorylation (Tyr705) but not total STAT3 was blocked by pifithrin-α treatment (Supplementary Figure 1). In addition, pifithrin-α treatment markedly suppressed the expression of collagen I, fibronectin, and α-SMA in UUO (Supplementary Figure 1).

### p53 knockout attenuated the renal fibrosis and apoptosis in UUO mice

**Figure 4:**
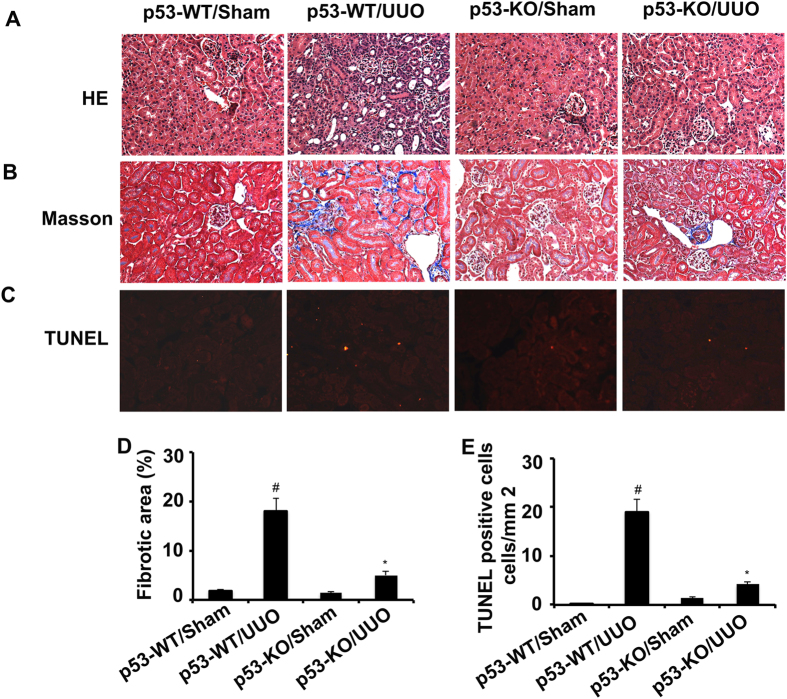
Both renal fibrosis and apoptosis were ameliorated in p53-KO mice. The left ureter of WT and p53-KO littermate mice was ligated for 7 days. Renal cortical tissues were collected for hematoxylin and eosin staining to examine histology (**A**), Masson staining for fibrosis analysis (**B**), and for TUNEL assay of apoptosis (**C**) and quantify tubulointerstitial fibrosis (**D**) and apoptosis (**E**) in the kidney cortex. Data were expressed as means ± sd (n = 6); ^#^*p* < *0.05*: the UUO group vs the sham group; *p < *0.05*: the PIF group vs the UUO group. Original magnification, x200.

We have demonstrated that pifithrin-α treatment ameliorated renal fibrosis in UUO mice. We further investigated whether genetic ablation of p53 played a protective role in UUO injury. The left ureter of p53-KO mice and their wild-type (p53-WT) littermates was ligated to induce renal fibrosis for 7 days. Histology, Masson trichrome staining and TUNEL analyses confirmed that UUO induced significantly lower tissue damage, fibrosis, and apoptosis in p53-KO mice compared with wild-type mice (Fig. 4A–E).

### p53 knockout reduced ECM accumulation and inflammation by blocking STAT3 activation in UUO mice

**Figure 5:**
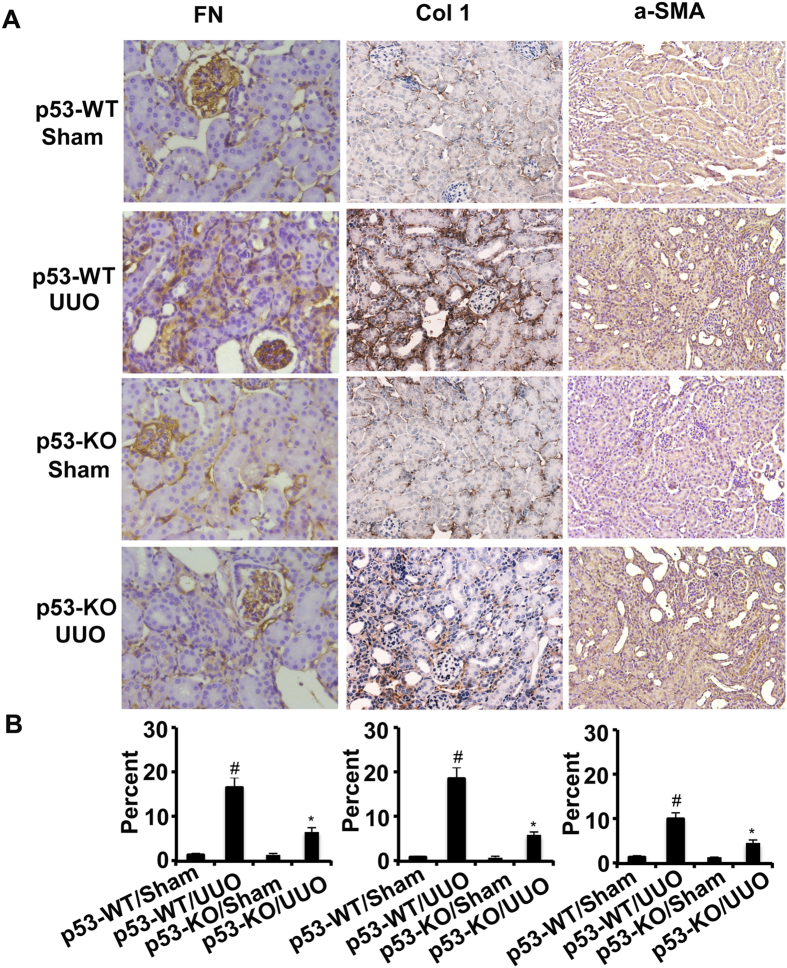
The expression of fibronectin, collagen I and α-SMA was suppressed by inhibition of STAT3 signaling in p53-KO mice. The left ureter of WT and p53-KO littermate mice was ligated for 7 days. Renal cortical tissues were collected for immunohistochemistry staining to examine expression of fibronectin, collagen I and α-SMA (**A**) and quantify immunohistochemistry staining (**B**). Data were expressed as means ± sd (n = 6); ^#^*p* < *0.05*: the UUO group vs the sham group; *p < *0.05*: the PIF group vs the UUO group. Original magnification, x200.

**Figure 6:**
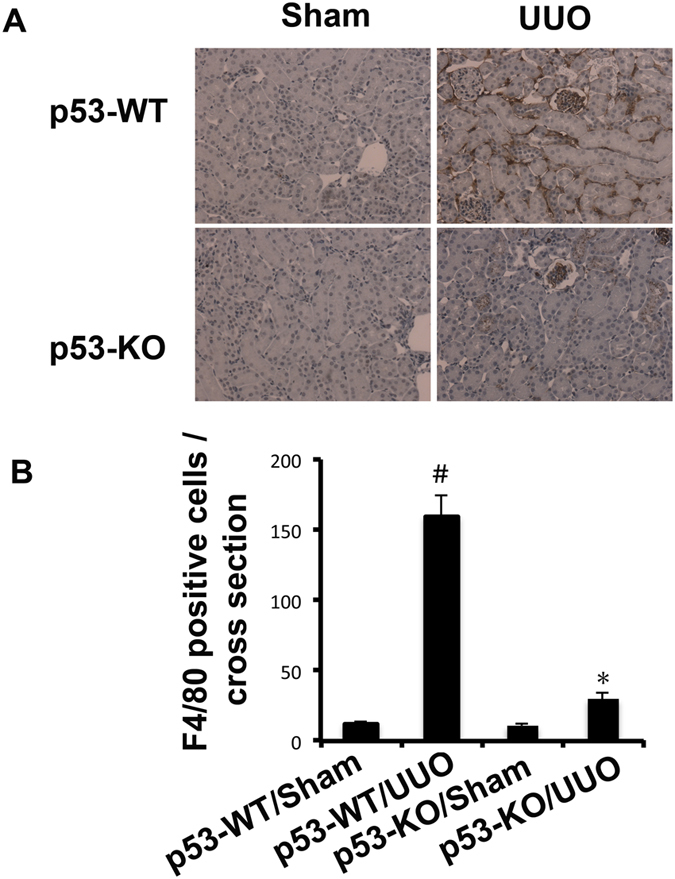
The infiltration of macrophage was suppressed in p53-KO mice. The left ureter of WT and p53-KO littermate mice was ligated for 7 days. Renal cortical tissues were collected for immunohistochemistry staining to examine expression of macrophage (**A**) and quantify immunohistochemistry staining (**B**). Data were expressed as means ± sd (n = 6); ^#^*p* < *0.05*: the UUO group vs the sham group; *p < *0.05*: the PIF group vs the UUO group. Original magnification, x200.

We found that the expression of fibronectin, collagen I, and α-SMA was markedly increased in wild-type mice with UUO than in the Sham group on day 7, which was significantly reduced in p53-KO mice (Fig. 5A,B). p53 expression in UUO was notably lower in p53 KO kidney tissues than that in wild-type tissues, verifying p53 ablation in knockout mice. After UUO injury, kidney tissues of p53-KO also showed less STAT3 tyrosine phosphorylation (Tyr705), lower expression of fibronectin, collagen I, and α-SMA (Supplementary Figure 2). Previous results demonstrated that deletion of p53 might ameliorate inflammation in ischemic AKI. This line of result drove us to examine whether the infiltration of inflammatory cells were also reduced in p53-KO mice during UUO injury. As shown in Fig. 6, UUO induced the infiltration of macrophage into wild-type kidney tissues, which was markedly suppressed in p53 KO tissues. The decrease of inflammation in p53 KO mice was correlated with STAT3 inactivation. These results further provided strong evidence that p53 played a critical role in the UUO model.

### The induction of miR-215, miR-199a-5p&3p after UUO was suppressed in PT-p53-KO mice

**Figure 7:**
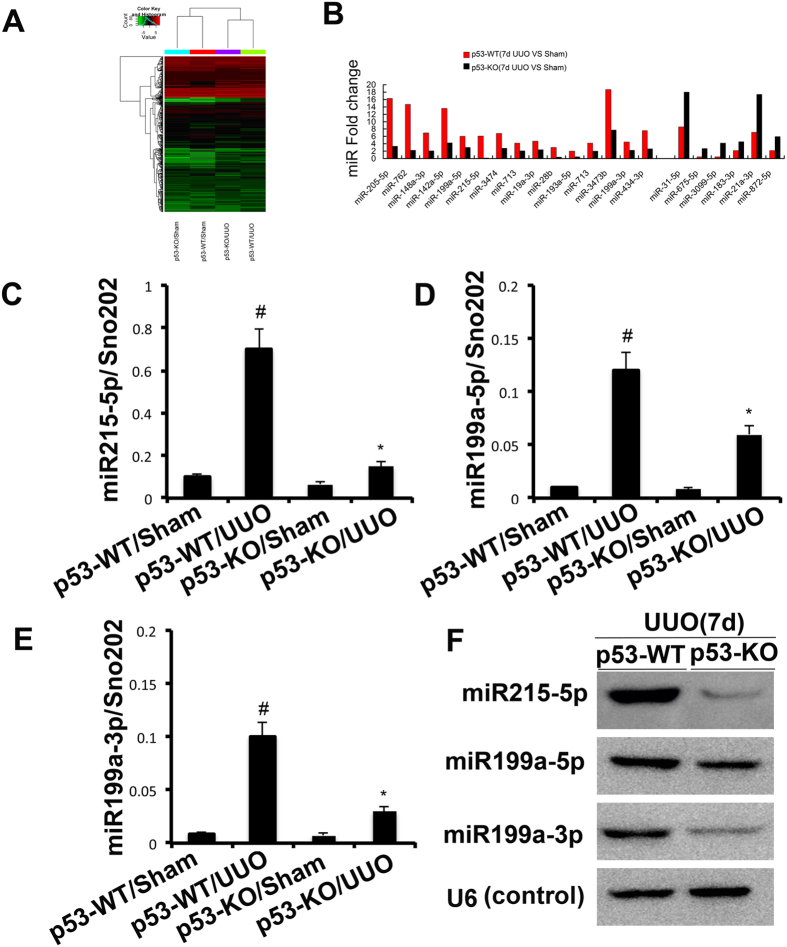
The induction of miR-215, miR-199a-5p, and miR-199a-3p after UUO was suppressed in PT-p53-KO mice. WT (n = 6) and p53-KO (n = 6) littermate mice with UUO were kept for 7 days or sham operation as control. Total RNA was isolated from renal cortical tissues of WT and p53-KO littermate mice. (**A**) Representative heat map of microRNA microarray analysis. The ΔCt values of all miRNAs were used to generate the heat map. (**B**) The amount of each miRNA from the UUO group was divided by the amount of sham control to calculate the fold change. (**C–E**) Real-time PCR analysis of miR-215-5p, miR-199a-5p&3p. The value of each miRNA was normalized by the signal of snoRNU202, an internal control. (**F**) Northern blot analyses of miR-215, miR-199a-5p, and miR-199a-3p. Total RNA (10 μg per lane) was analyzed by Northern blotting as described in the concise Methods section using a ^32^p-labeled probe of miR-215, miR-199a-5p, and miR-199a-3p. U6 was shown as an RNA loading control. Data were expressed as means ± sd (n = 6); ^#^*p* < *0.05*: the UUO group vs the sham group; *p < *0.05*: the PIF group vs the UUO group.

Both Zhu *et al*.^18^ and our present results demonstrated that inhibition of p53 activity blocked the STAT3 activation. However, the mechanism remains unclear. We hypothesized that miRNAs might be the mechanistic link between the induction of p53 activity and the activation of STAT3. A representative heat map of the microarray was shown in Fig. 7A. Among the 1153 detectable miRNAs, 15 miRNAs were downregulated in p53-KO tissues. However, 6 miRNAs were upregulated in p53-KO tissues (Fig. 7B). Of note, upregulated miR21 was related to the progression of renal fibrosis^19^. However, the two down-regulated miRNAs, miR-215-5p and miR-199a-5p were correlated with the amelioration of renal fibrosis^20^,^21^. MiR-199a-3p&5p were mature forms of mmu-miR-199a, hence, we further presumed that miR-199a-3p could be involved in the regulation of renal fibrosis like miR-199a-5p. The expression of miR-215-5p, miR-199a-5p & 3p was analyzed by real-time PCR and Northern blot (Fig. 7C–F), demonstrating that the induction of miR-215, miR-199a-5p&3p after UUO was inhibited in PT-p53-KO mice.

### MiR-199a-3p suppressed SOCS7 for STAT3 activation and renal fibrosis

**Figure 8:**
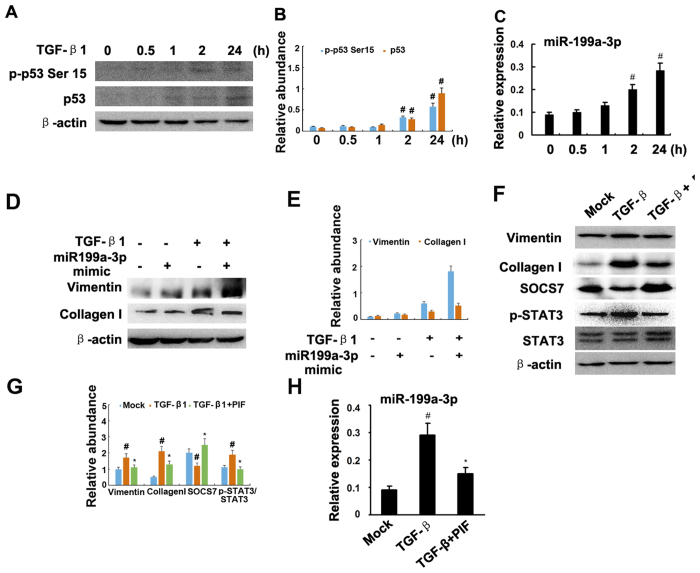
Pifithrin-α suppressed TGF-β1-induced STAT3 activation and miR-199a-3p expression. Cultured HK-2 cells were treated with 10 ng/ml TGF-β1 or 10 μM pifithrin-a for 0 to 24 h or transfection of miR-199a-3p or negative control analog or SOCS7 siRNA, followed by immunoblot for p-STAT3, STAT3, and ECM genes, and real time PCR for miR-199a-3p. Immunoblot (**A**) analysis of p-p53 Ser^15^ and p53, densitometry (**B**) of proteins signals on immunoblots, and real time PCR analysis (**C**) of miR-199a-3p after indicated time point of treatment with TGF-β1. Relative protein levels (**D**) of vimentin, COL1, and β-actin 24 h after transfection of miR-199a-3p analog (100 nM) or miR analog negative control (miR-ANC) with or without TGF-β1 treatment, densitometry (**E**) of proteins signals on immunoblots. Immunoblot analysis (**F**) of vimentin, COL1, SOCS7, p-STAT3, STAT3 and β-actin, densitometry (**G**) of proteins signals on immunoblots, and real time PCR analysis (**H**) of miR-199a-3p 24 h after TGF-β1 alone or TGF-β1 plus pifithrin-a treatment. Data were expressed as means ± sd (n = 6); ^#^*p* < *0.05*: 2 h or 24 h vs 0 h, or TGF-β1 group vs mock group; *p < *0.05*: TGF-β1 + PIF group vs TGF-β1 group.

**Figure 9:**
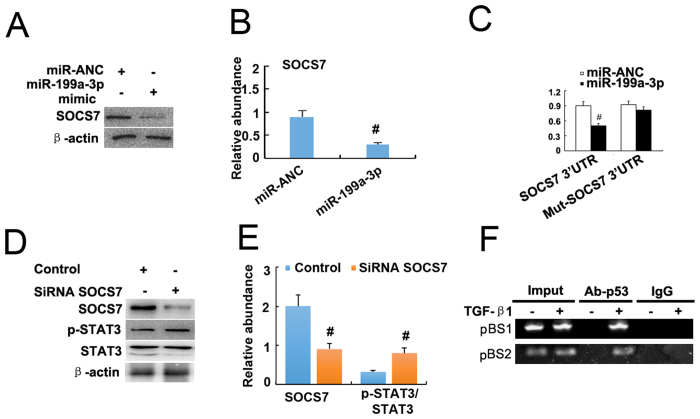
MiR-199a-3p suppressed SOCS7 to increase STAT3 activation. Cultured HK-2 cells were treated with transfection of miR-199a-3p or negative control analog or SOCS7 siRNA, followed by immunoblot for p-STAT3, STAT3, and ECM genes, and immunoprecipitation with antibodies to p53. (**A**) Relative protein levels of SOCS7 and β-actin 24 hours after transfection of miR-199a-3p analog (100 nM) or miR-ANC, densitometry (**B**) of proteins signals on immunoblots. (**C**) Detected luciferase activity 24 hours after cotransfection of miR-199a-3p analog (100 nM) or miR-ANC with SOCS7 3 ’UTR luciferase reporter vector. (**D**) Relative protein levels of p-STAT3 (Tyr705) and STAT3 24 hours after the transfection of SOCS7 siRNA or siRNA-NC, densitometry (**E**) of proteins signals on immunoblots. (**F**) ChIP assays for p53 were performed with chromatin material isolated from HK2 cells treated with TGF-β1. Precipitated DNA was amplied with oligonucleotides spanning regions of the potential p53 binding sites1 and 2 (pBS1and pBS2); total inputs were indicated. The antibody against p53 immunoprecipitated the DNA fragments from HK2 cells containing the potential pBS1and pBS2. Data were expressed as means ± sd (n = 6); ^#^*p* < *0.05*: miR-199a-3p vs miR-ANC, or SiRNA SOCS7 vs control group.

Jessica *et al*. have reported that TGF-β1 regulated p53 activity in human renal tubular epithelial cells (HK-2)^7^,^8^. In the present study, we further confirmed that TGF-β1 induced p53 accumulation and p53 Ser^15^ phosphorylation at different time point (Fig. 8A,B). Previous studies have demonstrated that both miR-215-5p and miR-199a-5p were related to the renal fibrosis^20^,^21^, hence, we focused on the miR-199a-3p. First, the real time PCR results demonstrated that miR-199a-3p was significantly induced by TGF-β1 at 2 h and 24 h (Fig. 8C). How about the role of miR199a-3p in the progression of fibrosis? The results indicated that TGF-β1 treatment markedly increased expression of vimentin and collagen I, which was significantly enhanced by transfection with miR-199a-3p analog (Fig. 8D,E). However, the regulation mechanism of miR-199a-3p for fibrosis remains unclear. We identified SOCS7 as the target of miR-199a-3p but not 5p predicted by the TargetScan database (http://www.targetscan.org/cgi-bin/targetscan/vert_61/view_gene.cgi?taxid=%2010090&rs=NM_014598&members=miR-199ab-3p/3129-5p&showcnc=1&shownc=1&showncf=1). We proposed that miR-199a-3p could promote STAT3 activation by inhibiting SOCS7. We found that TGF-β1 treatment markedly increased activation of STAT3 and the expression of collagen I, vimentin, and miR-199a-3p, and reduced SOCS7 protein, which was significantly reversed by pifithrin-α treatment (Fig. 8F–H). As shown in Fig. 9A,B, transfection of HK2 cells with miR-199a-3p also led to significant decrease in SOCS7 protein. To provide more direct evidence of miR-199a-3p targeting SOCS7, we co-transfected HK2 cells with a plasmid containing a luciferase report gene under the control of SOCS7 3′ untranslated region (UTR) and either a miR-199a-3p analog or a miRNA analog negative control. Luciferase activity was markedly reduced at 24 h after transfection in the presence of miR-199a-3p analog, compared with the negative control (Fig. 9C). Noguchi *et al*. reported that SOCS7 suppressed STAT3 activation in bladder cancer cells^22^. In HK-2 cells, we also found that p-STAT3 but not STAT3 was increased at 24 h after transfection with SOCS7 siRNA compared with the negative control (Fig. 9D,E). Furthermore, we used a ChIP assay to determine the interaction of p53 with the miR-199a-3p promoter region in HK-2 cells. As shown in Fig. 9F, the antibody directed against p53 immunoprecipitated the DNA fragments from HK-2 cells containing the potential binding sites of pBS1 and pBS2, supporting the hypothesis that p53 can physically interact with the miR-199a-3p promoter region. The results revealed that p53 induced miR-199a-3p to suppress SOCS7 for STAT3 activation.

### p53 knockout from proximal tubules attenuated renal fibrosis, apoptosis, and inflammation in UUO model

**Figure 10:**
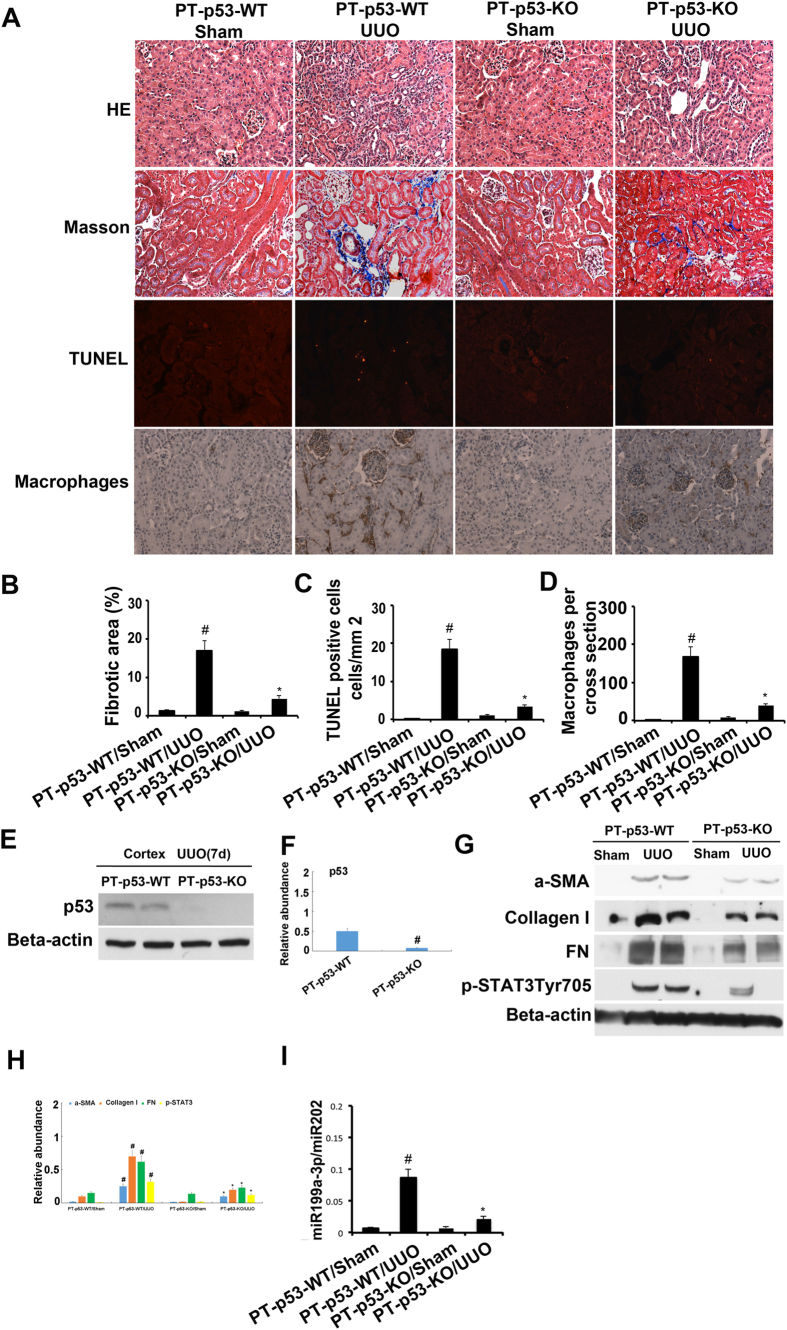
p53 deletion from proximal tubules attenuated renal fibrosis, apoptosis and inflammation in UUO-induced kidney injury. The left ureter of male proximal tubule–specific p53-knockout (PT-p53-KO) mice and wild-type (PT-p53-WT) was ligated for 7 days. (**A**) Renal cortical tissues were collected for hematoxylin and eosin staining to examine histology, Masson staining for fibrosis analysis, TUNEL assay of apoptosis, and for immunohistochemistry staining to examine expression of macrophages. Quantification of tubulointerstitial fibrosis (**B**), apoptosis (**C**) and immunohistochemistry staining (**D**) in the kidney cortex was done. The lysate of the kidney cortex was collected for immunoblot analysis of p53, p-STAT3 (Tyr705), fibronectin, collagen I, α-SMA, and β-actin by using specific antibodies (**E**,**G**), densitometry (**F**,**H**) of proteins signals on immunoblots, and real time PCR analysis of miR-199a-3p (**I**). Data were expressed as means ± sd (n = 6); ^#^*p* < *0.05*: the UUO group vs the sham group; *p < *0.05*: the PIF group vs the UUO group. Original magnification, x200.

Recent studies have suggested that p53 in different cell types may have distinct roles in renal fibrosis^10^,^11^,^12^. To determine the cellular origin of p53 that contributes to renal fibrosis after UUO injury, we used the proximal tubule–specific p53-knockout mouse model (PT-p53-KO) that was reported in our recent work^13^,^14^. The left ureter of the PT-p53-KO mice and the wild-type (PT-p53-WT) littermates were ligated for 7 days to induce UUO model. The PT-p53-WT animals with UUO showed tubular atrophy, interstitial collagen accumulation, tubular cell apoptosis and inflammation on day 7, which was notably ameliorated in PT-p53-KO tissues (Fig. 10A–D). p53 expression in the cortex during UUO injury was notably lower in the PT-p53-KO kidney tissues than in the wild-type tissues, verifying p53 deletion from the proximal tubules in knockout mice (Fig. 10E,F). We found that the expression of fibronectin, collagen I, α-SMA, STAT3 tyrosine phosphorylation (Tyr705), and miR199a-3p during UUO injury was markedly reduced in PT-p53-KO kidney tissues than in the wild-type tissues (Fig. 10G–I). These results support a critical role of proximal tubular p53 in UUO injury.

### Increment of p53-regulated gene expression in human kidneys

**Figure 11:**
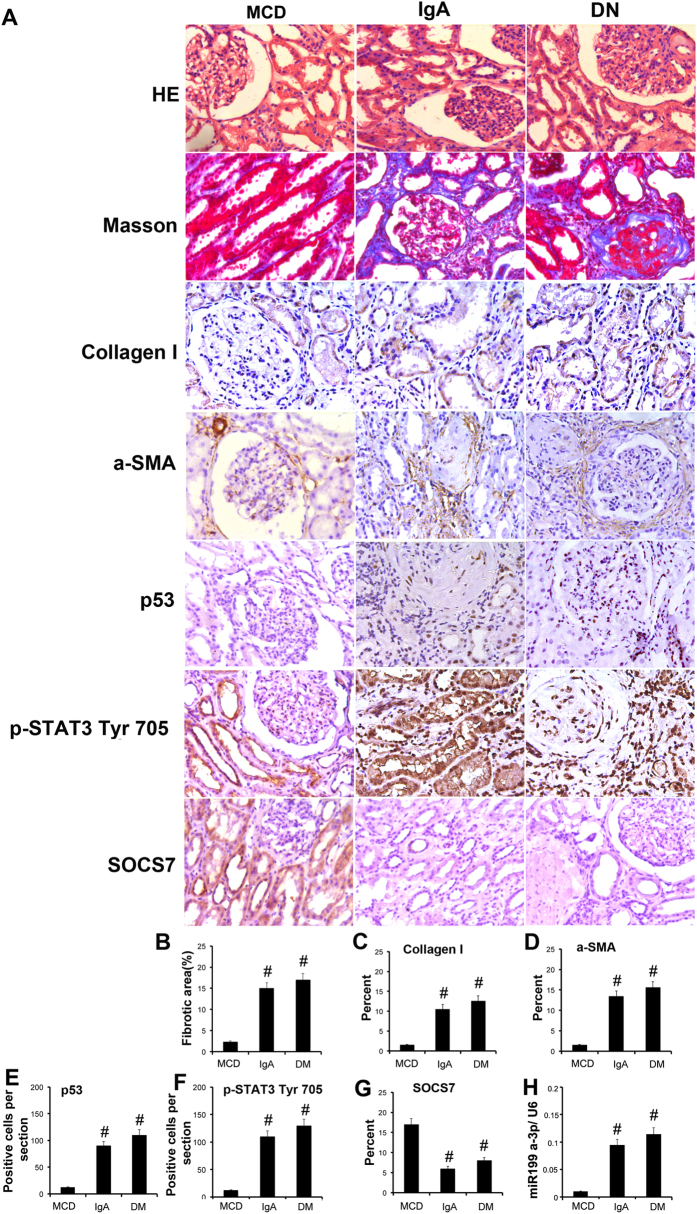
p53-induced related gene expression in patients with IgAN and DN. The kidneys from patients of IgAN and DN were collected for hematoxylin and eosin staining to examine histology, Masson staining for fibrosis analysis and immunohistochemistry staining for p53, ECM related gene expression, p-STAT3 and SOCS7 (**A**). Quantification of the tubulointerstitial fibrosis (**B**) in the kidney cortex. Quantify immunohistochemistry staining (**C–G**). Real time PCR analysis of miR-199a-3p (**H**). Data were expressed as means ± sd (n = 6); ^#^*P* < *0.05* versus MCD group. Original magnification, x200 or 400.

We corroborated the *in vitro* and *in vivo* findings by examining the renal expression of p53, ECM related gene expression, STAT3 and SOCS7 in severe IgA nephropathy (IgAN) and diabetes nephrology (DN). p53 and p-STAT3 nuclear staining, and expression of collagen I and α-SMA were markedly higher in kidneys of patients with IgAN and DN compared to kidneys from patients with minimal change disease (MCD) (n = 8 per group for each of the three groups) (Fig. 10A). However, we found that SOCS7 staining in IgAN and DN was notably lower than in kidneys with MCD. Consistent with these findings, tubulointerstitial injury or fibrosis was present in IgAN and DN kidneys but not in MCD kidneys (Fig. 11A). A semi-quantitative scoring of the immunostaining data was summarized in Fig. 11B–G. Furthermore, we detected that miR199a-3p expression in IgAN and DN was higher than in kidneys with MCD (Fig. 11H). These results further demonstrated that p53 regulated fibrosis in the human kidneys.

### The regulation mechanism of p53 in UUO-induced fibrosis

**Figure 12:**
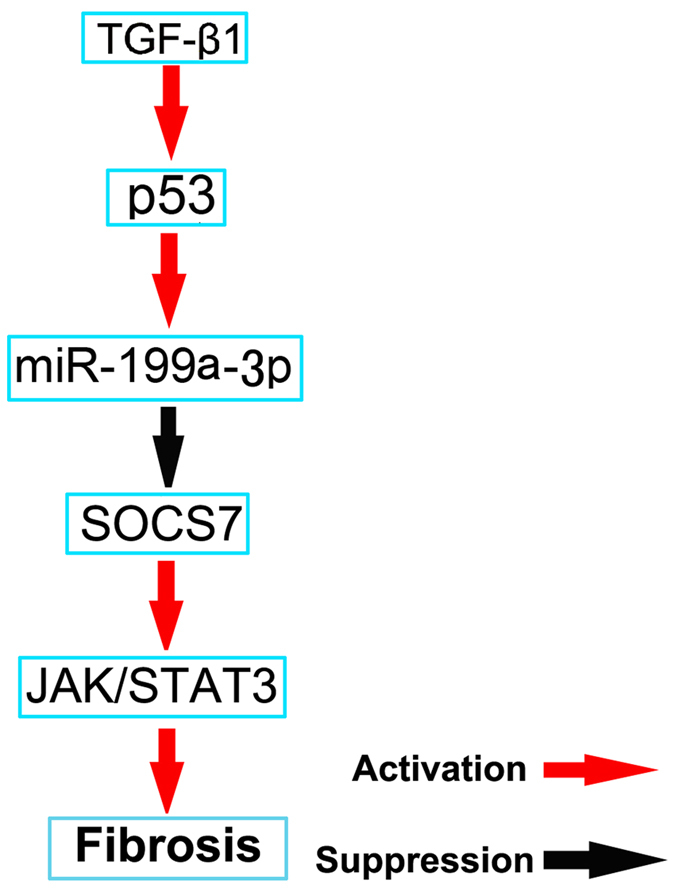
The role and molecular mechanism of p53 in UUO-induced renal fibrosis. TGF-β1 increased miR-199a-3p expression by induction of p53. Furthermore, miR-199a-3p directly suppressed SOCS7 expression, which led to activation of STAT3 and upregulation of the production of probrotic proteins.

We conclude the detail mechanism of p53-induced fibrosis (Fig. 12). TGF-β1 induced expression of p53, latter increased expression of miR-199a-3p to suppress SOCS7 expression and subsequently promote activation of STAT3 and ECM accumulation.

## Discussion

The pathologic role of p53 in renal fibrosis has been suggested. p53 inhibitors suppressed renal fibrosis *in post-ischemic kidneys*^11^. Moreover, the deletion of p53 from proximal tubules in mice reduced ischemic kidney injury and suppressed the associated interstitial fibrogenesis in the long term^10^. Our present study has further verified these observations by showing that p53 inhibitors and global or proximal tubular knockout of p53 ameliorated interstitial fibrosis during UUO in mice. Importantly, by the microarray analysis, we have identified three miRNAs that are upregulated through p53 in UUO. In addition, we have revealed that p53 induces STAT3 activation to promote renal fibrosis via direct upregulation of miR199a-3p to suppress SOCS7 in HK-2 cells. We have also detected consistent changes of these molecules in human kidney samples. Together, these results provide new insights into the mechanism of p53 regulation of renal fibrosis in relevant disease conditions.

p53 is a tumor suppressor that responds rapidly to genotoxic stresses induced by DNA damage, oncogene activation, hypoxia and reactive oxygen species in both cancer and normal cells. Upon activation, p53 may result in cell cycle arrest, apoptosis or cell death under various pathophysiologic conditions^23^. Our results demonstrate that p53 was mainly induced in the nuclei of renal tubular cells with some signal in the cytoplasm of a subset of tubular cells in UUO models, which was supported by previous observation that p53 was activated in the dysmorphic tubules of the obstructed kidney^8^. TUNEL assay showed a marked reduction of tubular apoptosis in the obstructed kidney by p53 inhibitors and global and proximal tubular knockout of p53 in mice, which is consistent with our previous findings that p53 was involved in renal tubular apoptosis in AKI models^13^,^14^. Renal tubular apoptosis during UUO may contribute to the accumulation of extracellular matrix surrounding the injured tubular cells and the progressive renal function loss^24^. As we know, TGF-β is also a key mediator in the progression of the process of fibrosis, which is activated and associated with fibrosis in UUO model^8^. Recent findings suggested that TGF-β induced p53 Ser15 phosphorylation and acetylation^25^,^26^. Consistently, our results demonstrate that TGF-β induced p53 accumulation and p53 Ser15 phosphorylation in HK-2 cells. Furthermore, our study has verified the injurious role of proximal tubular p53 in UUO. The role of p53 in the regulation of inflammation is complex. A recent study suggested that leukocyte p53 had a renoprotective role by its anti-inflammatory function by using chimeric mouse models^27^, while our previous study demonstrated that deletion of proximal tubular p53 suppressed inflammation in mice AKI model^14^. In our current study, renal interstitial inflammation was remarkably suppressed in the obstructed kidney of global and proximal tubular p53 knockout mice as demonstrated by the number of infiltrating macrophages into the kidney (Figs 6 and 9), which was correlated with lower apoptosis, supporting the finding that tubular apoptosis may drive inflammation^28^.

Previous results reported that TGF-β1 promoted SMAD3/p53 interactions to regulate renal fibrosis^7^. Recent research also reported that TGF-β1 also induced STAT3 activation^29^, which was detected in several human diseases associated with the progression of renal fibrosis, including glomerulonephritis and diabetic nephropathy^30^,^31^. Furthermore, STAT3 inhibitors ameliorated renal fibrosis in UUO^16^,^17^. In our present study, STAT3 activity was significantly blocked in the obstructed kidney by p53 inhibitors, and global or proximal tubular p53 knockout, which was supported by the evidence that p53 induced STAT3 activation in DOX-induced myocardial stress mode^18^. However, Lin *et al*. demonstrated that STAT3 was negatively regulated by p53 in prostate cancer cell lines^32^. These results indicate that the effect of p53 on STAT3 activity is dependent on the cell type.

To further investigate the mechanisms of p53 activity on the activation of STAT3 in the UUO model, microarray analyses were carried out using renal cortical tissues. The expressions of three miRNAs, miR-215-5p and miR-199a-5p&3p, were found to be consistently high in wide type mice with UUO, and they were decreased in renal cortex of global p53 knockout mice with UUO. Previous results demonstrated that p53 directly induced miR-215 expression^33^, which is known to be involved in increased collagen production and the progression of diabetic nephropathy by regulating the CTNNBIP1/β-catenin pathway^20^. miR-199a-5p (previously called miR-199a) and miR-199a-3p (previously called miR-199a*) are two mature forms derived from the same precursor in the human genome^34^,^35^. Numerous reports have now shown the involvement of these microRNAs in various types of tumors^36^,^37^,^38^. In the pathogenesis of tissue fibrosis, both miR-199a-5p and miR-199a-3p were associated with the progression of liver fibrosis in humans and mice^39^,^40^. Our data also demonstrate an enhanced renal expression of these two miRNAs in mouse UUO model. A recent study reported that miR-199a-5p suppressed caveolin1 for CCl4-induced liver fibrosis and UUO-induced renal fibrosis^21^. In current study, we found that miR-199a-3p was also a key effector of TGFβ signaling in HK-2 cells. Furthermore, our luciferase reporter assay identified SOCS7, one member of the family of suppressors of cytokine signaling (SOCS), as a target gene of miR-199a-3p in HK-2 cells. SOCS7-silencing in HK-2 cells significantly enhanced the STAT3 activation, which was supported by a previous study that SOCS-7 inhibited STAT3 activation by cytokine stimulation^41^. The mechanisms involved in the TGFβ-dependent modulation of miR-199a-3p are also of particular interest. A recent study reported that miR-199a-5p was regulated by TGF-β primarily through a Smad-dependent post-transcriptional mechanism promoting miRNA maturation by Drosha^42^. In the current study, by using ChIp assays, we demonstrated that p53 could physically interact with the promoter region of miR-199a-3p, which is supported by the work of Wang *et al*.^43^. Collectively, these data suggest a novel regulatory mechanism by which p53 upregulates STAT3 activation by direct inducing miR199a-3p to suppress SOCS7 in HK-2 cells (Fig. 11). This mechanism seems to be true not only in HK-2 cells, but also in mice and fibrotic kidney samples from IgAN and DN patients.

In conclusion, we have demonstrated that p53 plays a pivotal role in renal interstitial fibrosis in UUO, as evidenced by the alleviation of UUO-induced renal fibrosis by p53 inhibitors as well as global or proximal tubular p53 knockout in mice. In HK-2 cells and mouse models, inhibition of p53 suppressed STAT3 activation, a key signaling of renal fibrosis, by inducing miR199a-3p that represses SOCS7. Analysis of human kidney tissues provided evidence that p53/199a-3p /SOCS7/STAT3 axis may be involved in human renal fibrosis. Our present study suggests that p53 may be a therapeutic target of renal fibrosis in chronic kidney diseases.

## Materials and Methods

### Antibodies and reagents

Antibodies were obtained from different sources: anti-GAPDH, anti-β-actin, anti-α-SMA, anti-collagen І, and anti-fibronectin from Santa Cruz Biotechnology (Santa Cruz, CA, USA), macrophage from Abcam (Cambridge, MA, USA), polyclonal anti-p53, anti-phospho-p53-ser15, anti-STAT3, anti-p-STAT3 and SOCS7 from Cell Signaling Technology (Danvers, MA, USA). All secondary antibodies were from Thermo Fisher Scientiic (Waltham, MA, USA). Pifithrin-α was purchased from Sigma-Aldrich (Shanghai, China), the recombinant human TGF-β1 was obtained from R&D Systems (Minneapolis, MN, USA).

### Animals

C57BL/6J male mice were purchased from Shanghai Animal Center (Shanghai, People’s Republic of China). p53 global knockout mice were purchased from Shanghai Biomodel Organism Science & Technology Development Co., Ltd (Shanghai, People’s Republic of China). Proximal tubule-specific p53-deletion mice were produced by p53(flox/flox) mice (Jackson Laboratory) crossing with the PEPCK-Cre mice as described previously^13^,^14^. Use of animal and the experimental protocols were in accordance with the guidelines and approved by the Institutional Committee for the Care and Use of Laboratory Animals of Second Xiangya Hospital, People’s Republic of China. The mice were housed on a 12-hour light/dark cycle pattern with free access to food and water.

### Human samples

Human kidney samples were obtained from IgAN (n = 8), DN (n = 8), and MCD (n = 8). Parts of them were fixed with 4% buffered paraformaldehyde, and the others were soaked in RNAlater solution (Ambion), and then stored at −80 °C until use. Archival human kidney biopsies were collected at the Second Xiangya Hospital. Our study was approved by the Ethical Committee of Second Xiangya Hospital of Central South University (Changsha, China), and conducted in accordance with the relevant guidelines and regulations. Written informed consent was obtained for each participant.

### Animal model

The UUO model was established in male C57 black mice that weighed 20–25 g as previously described^44^. For Pifithrin-a treatment, four groups of mice comprising eight animals each (total 32) were divided into 4 groups: 1) Sham group with saline, 2) Sham with a dose of 3 mg/kg·d Pifithrin-α, 3) UUO group with saline, and 4) UUO with Pifithrin-α group.

### Cell culture and treatments

HK-2 cells were cultured in Dulbecco’s modified Eagle’s medium (Sigma-Aldrich) supplemented with 10% fetal bovine serum, 0.5% penicillin, and streptomycin in an atmosphere of 5% CO_2_ and 95% air at 37 °C. For Pifithrin-a treatment, HK-2 cells were treated with or without Pifithrin-a (10 μM) or TGF-β1 (10 ng/ml) for 24 h. For transfection experiment, after 24 h transfection of miR-199a-3p analog (100 nM) or negative control (miR-neg, Sigma) or SOCS7 siRNA (sc-41004, Santa Cruz, CA, USA). The culture media were changed regularly until cell confluency reached ~80%, followed by cell starvation in a serum-free medium overnight. They were then treated with or without either 0.1% BSA (control) or TGF-β1 (10 ng/ml) for another 24 h.

### Histology, Immunohistochemistry and Immunoblot Analyses

Kidney tissues were fixed with 4% buffered paraformaldehyde, embedded in paraffin, and 4 μm thick sections were prepared to staining with hematoxylin-eosin and Masson’s trichrome^44^. Immunohistochemical analyses were performed using p53, antiphospho-STAT3, anti-α-SMA, anti-collagen I, anti-fibronectin, anti-SOCS7 and anti-F4/80 or macrophage according to the previous protocol^14^. TUNEL assay was performed with the *In Situ* Cell Death Detection Kit from Roche Applied Science. For quantitation, the details were described in our recent work^17^. Briefly, 10–20 fields were randomly selected from each tissue section to count the TUNEL-positive cells per millimeter^14^. For immunoblot analysis, tissue lysates from kidneys or HK-2 cells were extracted for SDS polyacrylamide electrophoresis (Sigma-Aldrich) containing phosphatase inhibitors (Calbiochem). Blotting and antibody exposure were done by standard procedures.

### Microarray

The miRNAs were extracted from kidney cortical tissues for reverse transcription using the AmbionWT ExpressionKit (Life Technologies). MiRNA microarray procedures were performed at Shanghai Kangcheng Biological Corporation. Briefly, isolated miRNA was labeled using miRCURYTM Array Power Labeling kit (Exiqon). Labelled samples were hybridized to dual-channel microarrays. Scanning was performed with the Axon GenePix 4000B microarray scanner. GenePix pro V6.0 was used to read the raw intensity of the image. The data was analysed as described previously^45^,^46^. Successful array submission was made to the MIAMExpress.

### Real-Time PCR Analysis of miRNAs

Total RNA was extracted from kidney cortical tissues or HK-2 cells using the mirVana miRNA isolation kit (Applied Biosystems/Ambion, Austin, TX) according to the manufacturer’s instruction. Forty nanograms of total RNA was reverse-transcribed to cDNA using miRNA qRT-PCR Detection Kit (Ambion). Real-Time PCR was carried out using the Taqman miRNA assay kit (Applied Biosystems), including the sequence-specific primers for cDNA synthesis and Taqman probes for real-time PCR. Quantification was done using ΔCt values.

### Northern Blot Analysis of miRNAs

Total RNA was extracted using the mirVana miRNA isolation kit. Ten micrograms of RNA was run on a denaturing 10% polyacrylamide gel. The RNA was then transferred onto the Hybond-N+ membrane (Amersham, Piscataway, NJ), subjected to UV light irradiation for 4 min and baked at 80 °C for 1 h. The membrane was pre-hybridized for 1 h using ULTRAhyb-Oligo Hybridization Buffer (Applied Biosystems/Ambion) and subjected to hybridization with ^32^p-labeled antisense specific miRNA probe overnight at 37 °C. Then the membrane was washed in 2 × SSC buffer (0.1% SDS) and exposed to x-ray film at −80 °C.

### ChIP analysis

ChIP was performed as described previously^46^,^47^ with primary antibodies against p53. Precipitated DNAs were detected by PCR using specific primers: pBS1: 5′-CCACCCTCTTAG ATGCCTCA-3′ and 5′-CACTGGGGAAAGGCAGAG-3′, pBS2: 5′-ACCATGCTGAGCTCCTAACG-3′, and 5′-AGTTCAGGAGCAGCCACAGT-3′.

### Statistical Analyses

Quantitative data are expressed as means ± SDs. Quantitative data included immunoblots and tissue histology images, and represented at least three independent experiments. Statistical analysis was conducted using the Graph Pad Prism software. Multiple groups were compared by use of one-way analysis of variance followed by Tukey’spost-tests. Two-tailed unpaired or paired t tests were used to compare the differences of two groups. *P *<* 0.05* was considered significantly different.

## Additional Information

**How to cite this article:** Yang, R. *et al*. p53 induces miR199a-3p to suppress SOCS7 for STAT3 activation and renal fibrosis in UUO. *Sci. Rep.* **7**, 43409; doi: 10.1038/srep43409 (2017).

**Publisher's note:** Springer Nature remains neutral with regard to jurisdictional claims in published maps and institutional affiliations.

## Supplementary Material

Supplementary Information
