# Supplementary material for: p53 induces miR199a-3p to suppress SOCS7 for STAT3 activation and renal fibrosis in UUO
Source: Sci Rep. 2017 Feb 27;7:43409. doi: 10.1038/srep43409 (PMC5327480; doi:10.1038/srep43409)
Supplement: Supplementary Information [file srep43409-s1.pdf]

p53 induces miR199a-3p to suppress SOCS7 for STAT3 activation and renal fibrosis in UUO

Ruhao Yang, Xuan Xu, Huiling Li, Jinwen Chen, Xudong Xiang, Zheng Dong, Dongshan Zhang

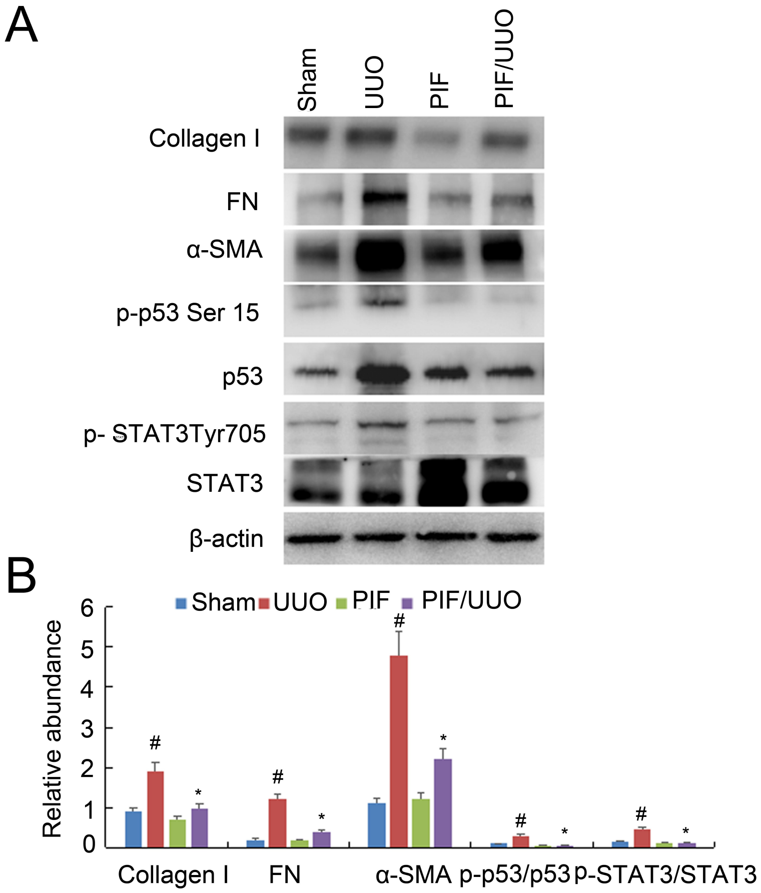

Supplementary Figure1. Pifithrin-a inhibits expression of collagenI, fibronectin and α-SMA by blocking STAT3 signaling in UUO mice. The male C57 mice after UUO were injected with 3 mg/kg pifithrin-a for 7 days. (A) The lysate of kidney cortex was collected for immunoblot analysis of collagen I, fibronectin, α-SMA, p-p53 (ser15), p53, p-STAT3 (Tyr705), STAT3, and β-actin by using specific antibodies. (B) densitometry of proteins signals on immunoblots. Data were expressed as means±sd (n=8); #*P<0.05* versus the sham group; * *P<0.05* versus the UUO group.

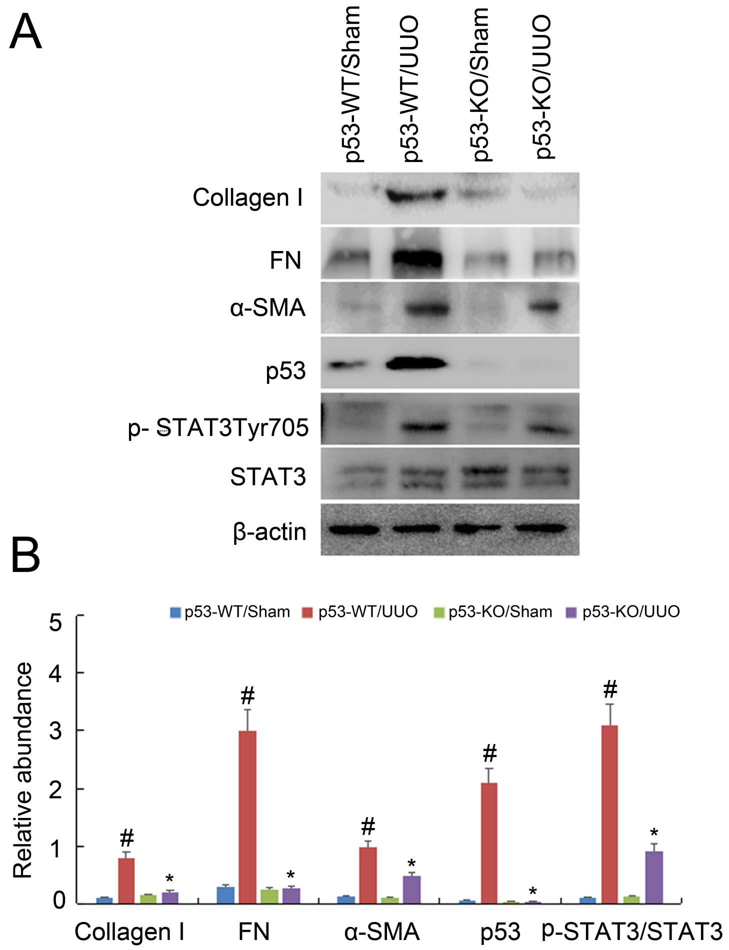

Supplementary Figure2. The expression of collagenI, fibronectin and α-SMA was suppressed by inhibiting STAT3 signaling in p53-KO mice. The left ureter of WT and p53-KO littermate mice was ligated for 7 days. (A) The lysate of kidney cortex was collected for immunoblot analysis of collagen I, fibronectin, α-SMA, p53, p-STAT3 (Tyr705), STAT3, and β-actin by using specific antibodies.(B) densitometry of proteins signals on immunoblots. Data were expressed as means±sd (n=6); #*P<0.05* versus the sham group; * *P<0.05* versus the UUO group.
